# Eight years post-Fukushima: is forest decontamination still necessary?

**DOI:** 10.1093/jrr/rrz047

**Published:** 2019-07-04

**Authors:** Yasuyuki Taira, Yudai Inadomi, Shota Hirajou, Yasuhiro Fukumoto, Makiko Orita, Yumiko Yamada, Noboru Takamura

**Affiliations:** Department of Global Health, Medicine and Welfare, Atomic Bomb Disease Institute, Nagasaki University, 1-12-4 Sakamoto, Nagasaki city, Nagasaki, Japan

Eight years have passed since the nuclear accident at the Fukushima Dai-ichi Nuclear Power Station (FDNPS), which is operated by the Tokyo Electric Power Company. Various radionuclides were released from the FDNPS into the atmosphere, eventually being deposited on land and at sea in the surrounding areas [1]. Following the accident, the residential areas, farmlands, forests (close to residential areas), and roads within the evacuation order areas around the FDNPS were extensively decontaminated by suitable methods, and decontamination of the entire area, excluding the difficult-to-return zones, was completed on 19 March 2018 [2]. Although radiation doses are decreasing in and around residential areas, it has been reported that forest areas accumulated 72% of the total atmospheric input of ^137^Cs in Fukushima Prefecture [3]. It has also been reported that radiocesium clearly accumulated in trees and the soil surface organic layer in the forest environment when the fallout occurred immediately after the FDNPS accident, but that this contamination decreased rapidly, resulting in a transfer of radioactivity to the mineral soil in the first 2 years after the FDNPS accident [4]. The mineral soil radiocesium inventory has not decreased significantly as a result of the balance between the decrease in the amount of radiocesium due to physical decay (longer half-life of ^137^Cs) and its migration from the soil surface organic layer above the mineral soil [4]. Moreover, the aerial deposition density in forest areas showed significant variability between municipalities because of multiple factors such as weathering processes and ecosystems [3]. Approximately 68% of Fukushima Prefecture is covered by forests, and residents frequently enjoyed the blessings of nature through outdoor activities such as hiking before the FDNPS accident [5]. Therefore, Fukushima residents desire further decontamination of forests from the perspective of forestry recovery and environmental preservation [6]. Actually, the forest area needing decontamination is extremely large and will require a huge budget [2]. Thus, from the perspective of cost-effectiveness and priorities, further discussions about forest decontamination policies are needed [7]. In the present study, we measured ambient dose equivalent rates (air dose rates) and analyzed the spectra of artificial radionuclides (mainly radiocesium) using a walking survey system (Radi-probe®; Chiyoda Technology Corp., Tokyo, Japan) and personal dosimeter (D-Shuttle®; Chiyoda Technology Corp.) to evaluate the contributory exposure doses to radiation (external exposure doses) and amount of environmental contamination in forest areas in Fukushima Prefecture [8–10].

**Fig. 1.**
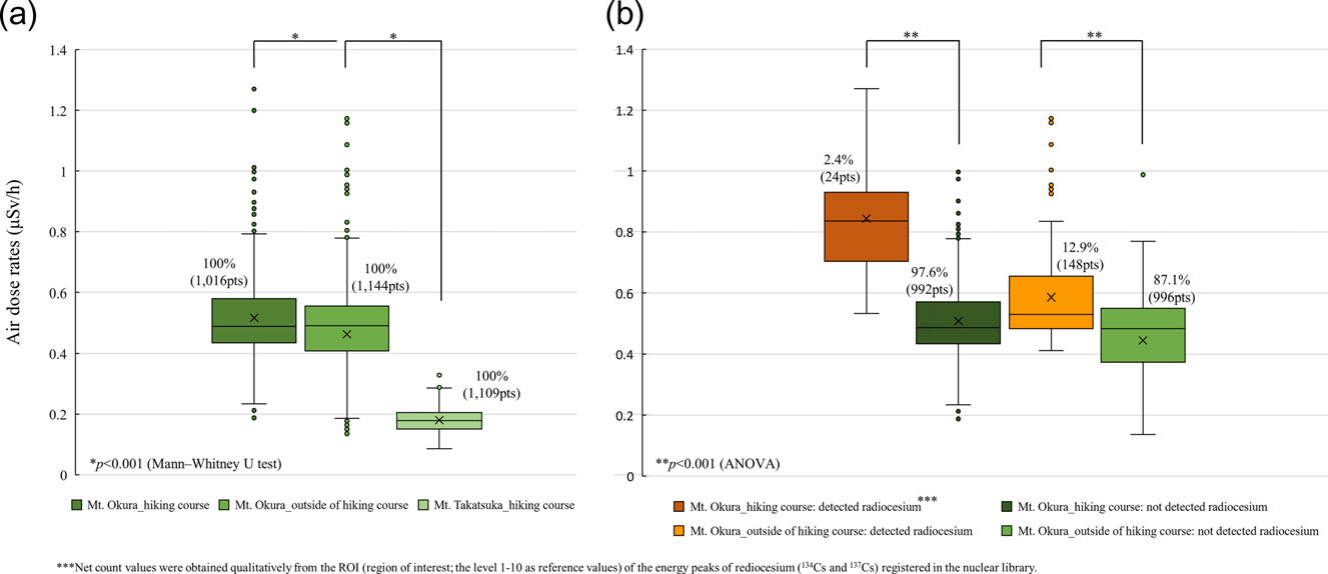
Distribution of air dose rates in the forest areas around Tomioka Town (October 2018) and Kawauchi Village (November 2018), Fukushima Prefecture. (**a**) All areas around Mt Okura. (**b**) Radiocesium-detected and non-detected areas around Mt Okura.

We measured the distribution of air dose rates in forests around Mt Okura (within and outside of the hiking course) in Tomioka Town, which is located 13.1 km southwest of the FDNPS, in October 2018, and around Mt Takatsuka (within the hiking course) in Kawauchi Village, which is located 29.5 km west-southwest of the FDNPS, in November 2018 (Fig. 1). At Mt Okura (Tomioka Town), the median air dose rates were 0.49 [0.19–1.3] μSv/h within the hiking course and 0.49 [0.14–1.2] μSv/h outside the hiking course. On the other hand, the median air dose rates within the hiking course at Mt Takatsuka (Kawauchi Village) were significantly lower (0.18 [0.086–0.33] μSv/h within the hiking course; *P* < 0.001). At Mt Okura, the median air dose rates within the hiking courses in the ‘radiocesium (^134^Cs and/or ^137^Cs)-detected areas’ and ‘radiocesium non-detected areas’ were significantly higher (0.84 [0.53–1.3] μSv/h and 0.49 [0.19–1.0] μSv/h, respectively; *P* < 0.001). In addition, the median air dose rates outside the hiking courses in the ‘radiocesium detected areas’ and ‘radiocesium non-detected areas’ were 0.53 [0.41–1.2] μSv/h and 0.48 [0.14–0.99] μSv/h, respectively (*P* < 0.001).

Moreover, the external exposure doses as measured by the personal dosimeter within and outside the hiking courses at Mt Okura were 0.88 μSv/2 h (0.42 mSv/year in case that the leisure time of Japanese workers is 20 h on Sunday) and 1.3 μSv/2 h (0.62 mSv/year), respectively. On the other hand, the median external exposure dose within the hiking course at Mt Takatsuka was 0.31 μSv/2 h (0.15 mSv/year) [11].

These findings indicate that the current radiocesium contamination levels differ substantially between Mt Okura and Mt Takatsuka, as radiocesium derived from the FDNPS accident is found in significantly higher levels at Mt Okura. The customs of residents, especially the ‘*satoyama*’ (countryside) culture of ingesting ‘*sansai*’ (edible wild plants), also require consideration in the further reconstruction of areas that were affected by the nuclear disaster such as Tomioka Town and Kawauchi Village [10, 12]. Although radiocesium contamination has been decreasing in the living spaces of Tomioka Town and Kawauchi Village, an external exposure risk remains in the forest environment [10]. Therefore, identifying decontamination areas (zoning) while managing external exposure and environmental contamination may help to recover the original landscape of Fukushima.
